# Personal Medicine and Bone Metastases: Biomarkers, Micro-RNAs and Bone Metastases

**DOI:** 10.3390/cancers12082109

**Published:** 2020-07-29

**Authors:** Steven L. Wood, Janet E. Brown

**Affiliations:** 1 Department of Oncology and Metabolism, Medical School, Beech Hill Road, Sheffield S10 2RX, UK; 2 Department of Oncology and Metabolism, Weston Park Hospital, Whitham Road, Sheffield S10 2SJ, UK; J.E.Brown@sheffield.ac.uk

**Keywords:** bone metastatic cancers, biomarkers, personalised medicine, micro-RNA, proteins, bone microenvironment

## Abstract

Bone metastasis is a major cause of morbidity within solid tumours of the breast, prostate, lung and kidney. Metastasis to the skeleton is associated with a wide range of complications including bone fractures, spinal cord compression, hypercalcaemia and increased bone pain. Improved treatments for bone metastasis, such as the use of anti-bone resorptive bisphosphonate agents, within post-menopausal women have improved disease-free survival; however, these treatments are not without side effects. There is thus a need for biomarkers, which will predict the risk of developing the spread to bone within these cancers. The application of molecular profiling techniques, together with animal model systems and engineered cell-lines has enabled the identification of a series of potential bone-metastasis biomarker molecules predictive of bone metastasis risk. Some of these biomarker candidates have been validated within patient-derived samples providing a step towards clinical utility. Recent developments in multiplex biomarker quantification now enable the simultaneous measurement of up to 96 micro-RNA/protein molecules in a spatially defined manner with single-cell resolution, thus enabling the characterisation of the key molecules active at the sites of pre-metastatic niche formation as well as tumour-stroma signalling. These technologies have considerable potential to inform biomarker discovery. Additionally, a potential future extension of these discoveries could also be the identification of novel drug targets within cancer spread to bone. This chapter summarises recent findings in biomarker discovery within the key bone metastatic cancers (breast, prostate, lung and renal cell carcinoma). Tissue-based and circulating blood-based biomarkers are discussed from the fields of genomics, epigenetic regulation (micro-RNAs) and protein/cell-signalling together with a discussion of the potential future development of these markers towards clinical development.

## 1. Introduction

Bone is the most common site of metastasis for many solid tumours (in particular breast and prostate cancer) [1]. Cancer spread to the skeleton is associated with a range of disease complications—termed skeletal-related events (SREs) including pathological fractures, spinal cord compression, severe bone pain and hypercalcaemia [1,2]. SREs cause a significant decrease in patient quality of life as well as resulting in a considerable economic burden on the healthcare system [3,4,5,6]—see Table 1.

Within breast cancer, metastases to the skeleton are detected in approximately 75% of patients with advanced breast cancer [7]. Despite improvements in the treatment for breast cancer, at the point of development of bone metastasis, breast cancer is currently incurable such that the median survival time following the diagnosis of bone metastasis ranges between 12 and 53 months [7].

Prostate cancer is the second most commonly diagnosed male cancer with 1.3 million cases reported in 2018 [8,9]. Mainly due to improvements in treatment, 84% of men with prostate cancer survive for 10 years or more. Metastasis to the skeleton is observed in up to 80% of advanced prostate cancer patients [10]. In contrast to bone lesions arising from breast cancer, which have mixed osteolytic and osteoblastic phenotypes, prostate cancer lesions are predominantly osteosclerotic. The new bone deposited in prostate cancer bone lesions has an unusual structure and is weaker than normal bone resulting in an increased rate of fractures and SREs. There is also an increased rate of bone resorption within prostate cancer bone lesions [11].

Lung cancer also metastasises to bone with the subtype of lung cancer being key at the time of primary diagnosis, such that the small-cell subtype has the highest incidence of skeletal involvement at diagnosis compared to squamous cell tumours. At the time of autopsy, the incidence of bone metastasis is 30% for all four subtypes of lung cancer. Bone metastases arising from lung cancer are predominantly osteolytic in nature. In contrast to metastases from breast and prostate cancer, the survival time of patients with bone metastases from lung cancer is relatively short with most patients dying within 12 to 18 months and a 5-year survival rate of only 10–20% [12,13].

Renal cell carcinoma (RCC) is responsible for over 200,000 new cancer diagnoses per year, with the incidence increasing at around 2% per year in Europe and North America [8,9]. Men have almost twice the incidence of renal cancer as women [14]. Renal cell carcinoma (RCC) accounts for 80–90% of all renal malignancies with a 5-year survival rate of approximately 45% [15,16,17]. Sub-divisions of RCC include clear cell, papillary and chromophobe, with clear cell disease being by far the most common. Although nephrectomy can be highly curable when RCC is detected at an early stage, between 20% and 50% of individuals (depending on the reference population and literature source) present with locally advanced or metastatic disease [16]. In a third of patients, metastatic disease appears later during disease progression following nephrectomy so that, overall, the majority of patients diagnosed with RCC will develop distant metastases.

There have been substantial recent advances in treatments for bone metastasis, such as the use of bisphosphonate anti-resorptive drugs and the anti-RANKL antibody therapeutic Denosumab for treatment of breast cancer bone spread, and these have improved patient survival; however, there is a strong need for the discovery of biomarkers predictive of bone metastasis risk within patients. This is particularly true for the administration of bisphosphonates, which can cause side effects including osteonecrosis of the jaw (ONJ). Biomarkers predictive of bone metastasis risk in the case of breast cancer will enable personalised medicine initiatives, whereby the molecular composition of a patient’s tumour determines the targeting and choice of treatments, minimising unwanted side effects. Biomarkers with potential to influence patient treatment decisions have arisen from genomic studies (including amplification of the MAF-gene [18,19]), protein-based biomarkers including increased expression of the proteins CAPG, GIPC1 and DOCK4 [20,21,22] and circulating products of extracellular matrix remodelling including the C-terminal propeptide of type-I collagen [11]. Recently interest has focussed upon the role of epigenetic regulators of the tumour and bone micro-environmental interactions, in particular, micro-RNAs within bone metastasis [23], and their potential as circulating markers of bone metastasis. We aim to summarise in this chapter the recent developments within the field of biomarker discovery for prediction of bone metastasis risk and their potential to influence personalised medicine initiatives.

## 2. Bone Homeostasis and the Vicious Cycle

Bone homeostasis is maintained by the actions of several cell-types, most notably bone-forming osteoblasts and bone-resorbing osteoclasts [24,25,26]. A wide variety of cytokines and growth factors regulate this process. In particular, pre-osteoclasts are activated to become active osteoclasts in response to the action of macrophage colony-stimulating factor (MCSF) and the receptor activator of nuclear factor-ĸB ligand (RANKL) [27]. Osteoclast activation is triggered by the binding of RANKL to the receptor activator of nuclear factor-ĸB (RANK). There are additional growth factors which mediate osteoclast activation including interleukin-11 (IL-11), prostaglandin-E2 and parathyroid hormone-related protein (PTHrP) [28].

Metastatic cancer cells alter the balance of osteoblast and osteoclast activation to alter normal bone homeostasis. Breast and lung cancer, as well as RCC, predominantly stimulates bone degradation (osteoclastic metastases), whilst prostate cancer is predominantly bone-forming (osteoblastic). Despite being predominantly bone-forming, prostate cancer also results in an increased risk of bone fractures as the new bone laid down has an unusual weaker structure than normal bone.

**Figure 1:**
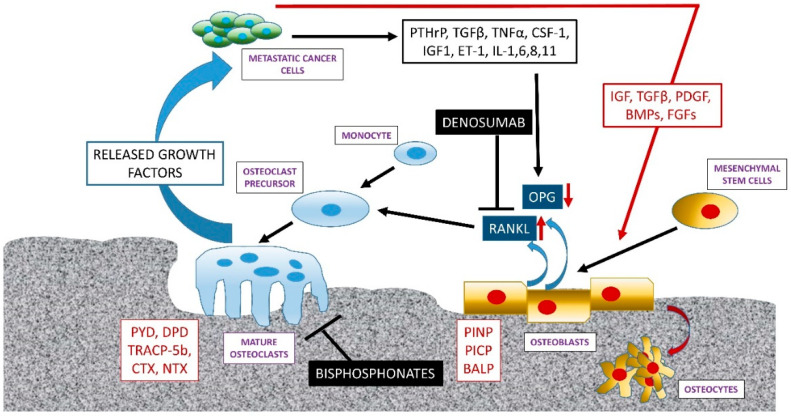
General mechanisms of altered bone homeostasis within bone metastatic cancers.

(i) Osteolytic Metastases:

Upon arrival in bone, metastatic breast, lung or RCC cancer cells secrete PTHrP which acts on osteoblasts to trigger an increased release of RANKL and reduced expression of the RANKL-antagonist osteoprotegerin (OPG). PTHrP is not the only growth factor released by metastatic breast cancer cells, which cause this alteration in RANKL: OPG, Wnt-family members, transforming growth-factor-β (TGFβ), endothelin-1 (ET-1) and bone morphogenetic proteins (BMPs) also play a key role. The increased expression of RANKL and reduced expression of OPG results in osteoclast activation and the consequent degradation of the bone matrix. Bone degradation releases trapped growth factors which then act upon the cancer cells to stimulate their proliferation resulting in a “vicious cycle” of bone destruction [26]—see Figure 1.

Bone turnover involves the action of bone absorbing osteoclasts and bone-forming osteoblasts. The actions of these two cell-types ensures normal bone homeostasis and continuous bone turnover. Mature osteoblasts are derived from mesenchymal stem cells, whilst mature active osteoclasts are derived from monocytes. Incoming metastatic cancer cells perturb this balance and alter bone turnover. Osteolytic cancers (breast, lung and RCC) secrete growth factors and cytokines (including PTHrP) which act on osteoblasts to increase their expression and secretion of RANKL, whilst decreasing their secretion of the RANKL decoy receptor OPG. The effect of this is to increase the binding of RANKL to RANK on osteoclast precursors and promote their activation. Mature, active osteoclasts degrade bone, releasing trapped growth factors, which then act on the metastatic cancer cells to promote their proliferation, in a “vicious cycle” of bone destruction. Osteoblastic cancers (prostate cancer) stimulate bone formation via promoting osteoblast differentiation via the release of growth factors and cytokines such as BMP, and TGFβ. Bone turnover results in the release of bone turnover markers (BTMs) including TRACP-5b, CTN and NTX (from bone degradation), and PINP, PICP and BALP from bone deposition. This system is the target of the current therapies bisphosphonates (targeting osteoclasts) and denosumab (binding and inhibiting RANKL).

Metastatic breast cancer cells extensively modify the extra-cellular matrix (ECM) in order to escape the primary tumour (extravasation), circulate in the bloodstream and eventually spread to bone. In order to do so, they actively secrete numerous matrix metalloproteinases (MMPs) and regulators of ECM remodelling. The diverse regulation of the MMP family of proteins within breast cancer bone metastasis is beyond the scope of this article, however, it has been reviewed extensively elsewhere [29,30].

In addition to the modification of the ECM, metastatic cancer cells also secrete other key regulators which play roles across multiple cancer types. Osteopontin (OPN) is one such regulator which plays a role in bone metastasis of both prostate and breast cancer. OPN (or secreted phosphoprotein-1) acts via binding to αvβ3 integrin and CD44 and plays a key role in both the metastatic spread as well as the drug-sensitivity of breast and prostate cancer. OPN is a promising target for therapeutic intervention within these cancer types [31].

(ii) Osteoblastic Metastases:

Prostate cancer metastasis to bone can induce osteolytic as well as osteoblastic lesions, with the osteoblastic phenotype being the more prevalent outcome. There is evidence that most of the tumour-derived growth factors acting within breast cancer are also integral to prostate cancer signalling [32]. Osteoblastic lesions arise from cancer-induced signalling via bone morphogenetic proteins (BMPs), fibroblast growth factor and TGFβ resulting in osteoblast activation [24]. Expression of BMP-2, -4, -6 and -7 is elevated within bone metastases [33] within which BMPs can induce the osteogenic differentiation of osteoblasts [34].

In addition to the extensive interaction of metastatic cancer cells with the bone micro-environment via either autocrine or paracrine signalling events, several genetic alterations have been discovered within the metastatic cancer cells themselves which account for their metastatic phenotype. These will now be discussed.

### 2.1. Breast Cancer

Genes which predispose breast cancer cells towards metastasis to bone have been characterised by employing copy number analysis (CNA) within breast cancer cell lines (MCF7 and MDA-MB-231) and osteotropic variants of these cell lines, isolated within murine models of breast cancer bone homing. Copy number analysis identified the amplification of the 16q32 chromosomal region within the osteotropic cell line with an average 1.5-fold increase in the number of copies per cell [19]. Sequencing of the 16q32 region identified the v-maf avian musculoaponeurotic fibrosarcoma (MAF) oncogene homolog within this region. The MAF-transcription factor regulates the expression of proteins including PTHrP, involved in the vicious cycle of bone destruction, and MAF amplification within primary breast cancer tumours significantly correlates with bone metastasis risk (HR = 14.5, CI = 6.4 to 32.9, *p* < 0.001) [18,19,35].

The identification of gene expression profiles which predict the risk of bone metastasis has also recently focused upon gene expression databases resulting in a proposed predictive gene expression nomogram [36]. Measuring the level of five bone metastases-related genes (KRT23, REEP1, SPIB, ALDH3B2 and GLDC) was observed to predict bone metastasis risk within both patient training and testing sets [36].

### 2.2. Prostate Cancer

Studies performing genome-wide expression studies (GWAS) within prostate cancer bone metastases from patients who were either untreated or subjected to androgen-deprivation therapy (ADT) have identified three distinct molecular subtypes within bone lesions [37]. These three subtypes termed MetA-C display unique gene expression profiles. The MetA subtype was the most abundant gene expression profile within bone metastases being responsible for 71% of cases and showing increased expression of androgen-receptor-regulated genes (including prostate-specific antigen—PSA). The other molecular subtypes observed classified as MetB and MetC were responsible for 17% and 12% of cases, respectively. MetB bone metastases have alterations in genes associated with DNA damage repair and cell cycle regulation, whilst MetC metastases have enrichment for genes involved in stromal-epithelial cell interactions [37]. These genetic profiles within bone metastases were observed to correlate with different therapeutic outcomes, in particular, MetB bone metastases had the lowest levels of serum PSA as well as the poorest outcomes following ADT [37].

### 2.3. Lung Cancer

Genetic mutations which predispose lung cancer patients towards the development of bone metastasis have been studied by the application of next-generation sequencing (NGS). Exome sequencing conducted within eight primary tumours from NSCLC patients, compared to comparable sequencing within lung cancer patients with bone metastasis, focussed on 483 tumour-associated genes [38]. This study identified mutations, insertions and deletions within genes, with the top three genes observed to be frequently mutated being fibroblast growth factor receptor (FGFR), ataxia telangiectasia mutated (ATM), and cyclin-dependent kinase-12 (CDK12) [38]. In addition, mutations were detected within all patients sequenced for hepatocyte nuclear factor 1 alpha (HNF1α), adenomatous polyposis coli (APC), and CD22 with an over 50% mutation frequency (being 75%, 62.5%, and 50%, respectively) suggesting that these may be the genes with the greatest potential to account for bone metastasis within lung cancer patients [38].

### 2.4. Renal Cell Carcinoma

To date, few studies have identified genetic mutations specifically associated with the metastasis of RCC to bone sites. Next-generation sequencing has been applied to RCC primary tumours and samples isolated from metastatic sites demonstrating considerable genetic variation between the two within individual patients [39]. In this study which performed sequencing for mutations within 341 selected genes, there were no mutations detected that were enriched in either the primary or metastatic sites [39]. There was a higher incidence of genetic discordance between primary and metastatic site samples in patients harbouring mutations within the SETD2 (SET domain containing 2, histone lysine methyltransferase) gene, suggesting that this gene may regulate the rate of genetic variation within RCC cells enabling the generation of cell lines harbouring pro-metastatic mutations [39].

## 3. Treatment of Bone Metastases

The treatment of bone metastasis arising from breast or prostate cancer currently involves the use of anti-resorptive agents, such as bisphosphonates (i.e., zoledronic acid) [40,41], Denosumab [42] and (in the case of prostate cancer) ^223^Radium [43], and these agents have become a central part of treatments used to reduce the impact of SREs within cancer patients. A complete description of the treatment options for bone metastatic cancers is beyond the scope of this review but has been described elsewhere [42,44,45,46]. Two bone-targeted agents have gained widespread clinical applicability within breast cancer and their mechanisms of action are relevant as they both highlight the key importance of tumour-cell interactions within the bone micro-environment, as well as the case for predictive biomarkers is evident owing to side effects within the treatments. These concerns and mechanisms are highly relevant to the treatment of all bone metastatic cancers.

### 3.1. Bisphosphonates

The bisphosphonate class of drugs include the “non-nitrogen containing” bisphosphonates (etidronate and clodronate) and the “nitrogen-containing” bisphosphonates (risedronate, zoledronate, alendronate and ibandronate) [47]. The chemical structure of bisphosphonates, consisting of a P-C-P group (instead of a P-O-P group) ensures their rapid uptake by bone. Following the administration of oral bisphosphonates, approximately 50% of the dose is excreted by the kidneys, with the remaining bisphosphonate being taken up by bone resulting in an effective concentration of drug of up to 1000 mM within the resorption lacunae [48,49]. Bisphosphonates are currently used in the treatment of metastases from breast and prostate cancer, as well as in the treatment of multiple myeloma.

Bisphosphonates induce osteoclast apoptosis thus reducing osteoclast-mediated bone destruction. In a recent meta-analysis of data from the large international randomised phase-III AZURE trial, looking at the addition of bisphosphonates to standard adjuvant therapy in the treatment of patients with stage II/III breast cancer, bisphosphonate addition was observed to improve DFS and IDFS within postmenopausal women (HR*_DFS_* = 0.82, 95%CI = 0.67–1.00; HR*_IDFS_* = 0.78, 95%CI = 0.64–0.94) [35]. Despite this utility, bisphosphonates are not without side effects, most prominently osteonecrosis of the jaw (ONJ) [50]. Although the actions of bisphosphonates are mainly attributed to their induction of osteoclast apoptosis, increasing evidence suggests that bisphosphonates may also prevent apoptosis of osteoblasts and osteocytes, as well as promote the proliferation of osteoblasts [51].

### 3.2. Denosumab

Additional therapies have been devised for the treatment of bone metastasis including the humanised anti-RANKL monoclonal antibody agent Denosumab [42]. Within breast and prostate cancer denosumab was observed to be superior to zoledronic acid in preventing the development of first SREs [52,53] and denosumab is currently used in the clinic to treat bone metastases from breast and prostate cancer. Denosumab acts to prevent osteoclast maturation (in contrast to bisphosphonates, which induce osteoclast apoptosis) and act to reduce bone resorption. To date, there have been no effects reported of denosumab upon osteoblasts. Denosumab has side effects which are similar to those associated with the bisphosphonate class of drugs, namely: fatigue, nausea, diarrhoea, and osteonecrosis of the jaw, with the latter being an equally rare complication [54].

Biomarkers are thus urgently required to predict which patients are at highest risk of developing bone metastasis, enabling targeted treatment for higher-risk patients.

## 4. Biomarkers for Bone Metastasis

There is a pressing need for biomarkers predictive of bone metastasis. The development of SREs, results in release of molecular fragments derived from pro-collagen cleavage, in particular, pro-collagen type-I N-terminal pro-peptide (P1NP) and pro-collagen type I C-terminal pro-peptide (P1CP) as well as pyridinoline (PYD) and deoxypyridinoline (DPD). These protein fragments can be measured in serum and some are also detectable in urine [11]. A detailed summary of the use of bone turnover markers (BTMs) within the treatment of SREs is beyond the scope of this review, however, they are summarised in [55] and outlined in Table 2. Despite their utility, BTMs suffer several limitations including the wide range of hormone therapies used in cancer patients which interfere with BTM levels (as well as patient-related features-such as age, sex, presence of kidney and/or liver disease) which alter BTM levels [56].

**Figure 2:**
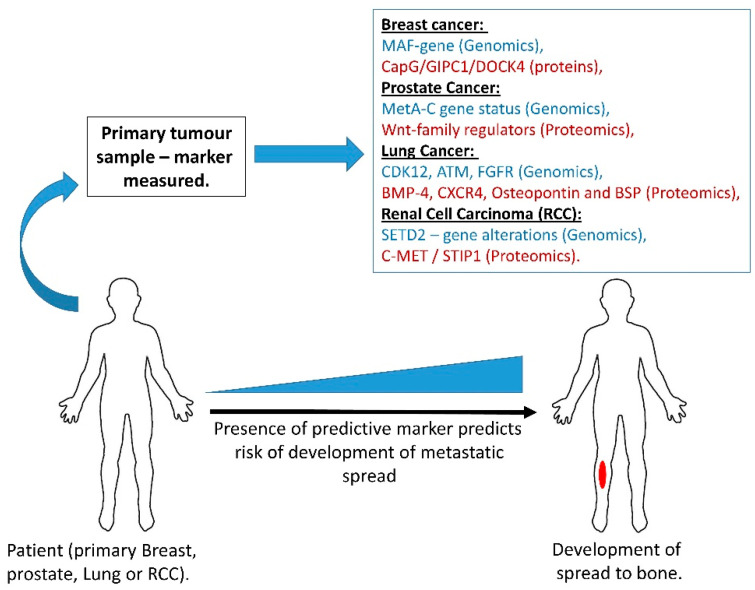
Tissue and potentially circulating biomarkers predictive of risk for cancer spread to bone.

Biomarkers are thus urgently required in the diagnosis of bone metastasis as well as in identifying patients at high risk of developing spread to bone—see Figure 2.

Biomarkers are required to predict the risk of primary tumours spreading to bone. This will enable the targeting of current bone-directed therapies towards those patients who will benefit the most, thus sparing patients any unwanted side effects. These markers can have a genetic origin or be micro-RNAs or protein-based and studies have identified potential biomarkers in all of these classes. Molecules which are released from tumours into the bloodstream have the greatest utility for longitudinal monitoring of patients due to their ability to be sampled non-invasively. Tissue-based markers, cannot be sampled non-invasively, however, they have greatly revolutionised the treatment of patients with bone-metastatic cancers.

There is also a requirement for biomarkers which will predict response to therapies (prognostic markers). The following sections outline current biomarker discovery studies within genomics, epigenetics (at the level of regulatory micro-RNAs) and proteins. The utility of these markers varies from study to study, some biomarkers predict the risk of developing bone metastasis, some markers have additional utility within the diagnosis of bone metastasis as well as informing patient-treatment decisions. The potential applications of the individual markers are listed in each case.

### 4.1. Micro-RNAs as Key Regulators of Metastasis

An extensive body of research has highlighted the pivotal role small non-coding RNAs (micro-RNAs) play in the regulation of diverse cellular processes including cell growth, differentiation, programmed cell death, apoptosis and the cell cycle [71]. Micro-RNAs are 18–25 nucleotide long RNAs which bind to the 3′ or 5′ untranslated region (UTR) of mRNAs resulting mostly in the degradation of the mRNA or the regulation of protein translation [72]. Micro-RNAs act via complementary binding to regions of sequence homology, but may also bind to sequences with partial homology, with the thermodynamic stability of the micro-RNA-mRNA complex being pivotal [73]. Due to this mode of action, each individual micro-RNA can regulate multiple mRNAs, and each mRNA can be the target of multiple micro-RNAs. Numerous studies have highlighted the key role of micro-RNAs in the regulation of cancer and metastasis [74,75,76,77,78].

Micro-RNAs can be isolated and quantified from a wide variety of biological sources, including tissue samples, blood-based samples, urine and circulating and secreted exosomes [79,80]. Due to their role in the regulation of the steps of metastasis, and the relative ease of detecting and measuring these molecules within biological samples, there has been great interest in the role of micro-RNAs as circulating biomarkers within cancer metastasis to bone. The main marker candidates predictive of bone metastatic outcomes within patients arising from genomic and protein-based studies are summarised in Table S1

#### 4.1.1. Breast Cancer

The absence of reliable biomarkers that predict the risk of developing bone metastasis within breast cancer patients has prompted considerable research into the potential of micro-RNAs as prognostic and diagnostic agents.

Studies conducted using both in vitro and in vivo approaches have identified the miR-30 family of micro-RNAs as being pivotal within bone metastasis of breast cancer. Metastatic spread of breast cancer cells to bone involves the increased expression of bone-related genes within the breast cancer cell. An integral player in this process of osteomimicry is the transcription factor RUNX2 [81]. Genes with increased expression mediated by RUNX2 including Collagen-α1, BSP, osteocalcin and osteopontin [82]. The expression of RUNX2 is regulated by a series of micro-RNAs including miR-30 family members (as well as miR-203 and miR-135a). Low expression of miR-30 within primary breast tumours is prognostic for poor relapse-free survival [83]. The role of miR-30 family members within bone metastasis was demonstrated by studies in which over-expression of miR-30 was observed to inhibit tumour growth in bone, reduce bone destruction and inhibit cancer cell invasion [83]. miR-30 levels correlated with ER/PR status, with ER/PR negative cells expressing lower levels of miR-30 family members than ER/PR positive cells [83]. The targets of RUNX2 which are responsible for osteomimicry are still being elucidated; however, studies have identified integrin-alpha-5 (ITGA5) and cadherin-11 (CDH11) as additional targets of miR-30 action, in addition to RUNX2.

Whilst studies have demonstrated the importance of micro-RNAs within bone metastasis, few have looked at the circulating levels of micro-RNAs within the bloodstream as a predictive tool. One such study is the elucidation of the role of miR-218 within breast cancer metastasis. Micro-RNA-218 levels are elevated within serum samples from patients with bone metastases compared to patients without metastatic spread [84]. Micro-RNA-218 is secreted by breast cancer cells within extracellular vesicles and mechanistically acts to inhibit expression of the proteins sFRP-2 and Sclerostin which are key inhibitors of Wnt-family signalling [85]. Activation of Wnt-family proteins occurs within breast cancer bone metastases, and key Wnt family targets involved in this process include PTHrP [85,86]. In this way, breast cancer secreted miR-218 can act to promote Wnt-mediated osteoclast differentiation and consequent bone destruction. Studies have demonstrated additional roles of miR-218 outside of promoting osteoclast activation, including uptake by osteoblasts inducing their reduced expression of collagen-I [84].

Several micro-RNAs have been implicated in the alterations in bone formation and breakdown induced by incoming metastatic breast cancer cells. The key role of miR-34a-5p within the inhibition of bone resorption has been demonstrated, and that this micro-RNA targets the pro-osteoclastogenic growth factor transforming growth factor-β-induced factor 2 (TGif2) [81]. Analysis within patient-derived samples demonstrated increased expression of miR-34a-5p within ductal carcinoma in situ (DCIS) samples compared to healthy mammary gland samples, as well as a greatly reduced expression within bone metastases [81].

#### 4.1.2. Prostate Cancer

Micro-RNAs play a key role in the regulation of prostate cancer spread to bone and regulate all the key steps of metastatic dissemination.

Comparison of the micro-RNA expression profiles between bone-metastatic prostate cancer tissues and non-bone metastatic prostate cancer demonstrated that reduced miR-141-3p expression correlated with spread to bone [87]. The role of miR-141-3p in prostate cancer bone metastasis is complex with widespread effects upon numerous targets. Targets of miR-141-3p include tumour necrosis factor receptor-associated factor 5 (TRAF5) and 6 (TRAF6), which act to inhibit nuclear factor-ĸB (NF-ĸB) signalling resulting in increased bone metastasis. In addition, miR-141-3p has also been implicated as a key regulator of epithelial–mesenchymal transition (EMT) within prostate cancer. The over-expression of miR-141-3p within prostate cancer cells has been demonstrated to inhibit their invasive and migratory capabilities, whilst a reduction in miR-141-3p levels was observed to promote the spread of prostate cancer to bone [87].

A key pathway active in the bone metastatic spread of prostate cancer is TGFβ signalling. Studies have demonstrated that miR-19a-3p expression inhibits bone metastasis via targeting the TGFβ-signalling effectors SMAD2 and SMAD4 [87]. Studies demonstrated a reduced expression of miR-19a-3p as prostate cancer cells develop bone homing ability, as well as within prostate cancer tissues from patients with bone metastases.

Genetic studies have identified a cluster of micro-RNAs located on chromosome 14q32 implicated in prostate cancer bone metastasis, including the delta-like-1 homolog—deiodinase, iodothyronine 3 (DLK1-DIO3) region encompassing miR-154* (an additional micro-RNA from the miR-154 hairpin), miR-379 and miR-409-3p/-5p, all upregulated within prostate cancer and correlated with cancer spread to bone [88,89,90]. Increased expression of these micro-RNAs is observed within animal models of prostate cancer bone metastasis, and the central role of these micro-RNAs has been further validated within patient-derived samples [91,92,93]. Some of these markers have demonstrated ability to act as circulating markers of prostate cancer bone metastasis, in particular, miR-409-3p which has increased expression within the serum of patients with high-risk prostate cancer compared to low-risk patients [84]. In addition to displaying potential as circulating markers, these micro-RNAs also have potential to be tissue-based biomarkers, as demonstrated by miR-154* and miR-409-3p/-5p which display a higher level expression within prostate cancer bone metastatic samples in comparison to localised prostate cancer tissue samples [88,89].

Comparison of tissue samples from prostate cancer patients with bone metastases, and normal prostate tissue sections, as well as serum samples, identified a key role for miR-218-5p in the prediction of prostate cancer bone-metastasis-free survival [94]. Measurement of circulating serum miR-218-5p levels in patients demonstrated a ROC-curve area under curve (AUC) of 0.86 (95% confidence interval 0.80–0.92, *p* < 0.001) in studies of bone metastasis DFS [94]. Studies within prostate cancer cells demonstrated that miR-218-5p inhibited NF-κB signalling by targeting TRAF1, TRAF2 and TRAF5 resulting in reduced invasive and migration abilities [94].

#### 4.1.3. Lung Cancer

Micro-RNAs have been implicated in the priming of bone sites for the arrival of metastatic lung cancer cells via promoting the conversion of mesenchymal stem cells (MSCs) into active osteoblasts. Exposure of MSCs to pro-osteogenic medium from NSCLC cells resulted in a decreased expression of miR-139-5p [95]. The action of miR-139-5p was demonstrated to occur via the downregulation of Notch-1 signalling within MSCs. Additionally, circulating serum levels of miR-139-5p were observed to be lower in lung cancer patients who developed bone metastases compared to patients with metastases to other sites [95].

Micro-RNAs have been implicated in the mode of action of genes, which act to regulate lung cancer metastasis. The gene Nm23-H1 is a demonstrated inhibitor of lung cancer metastasis to bone, and qRT-PCR-based studies demonstrated that Nm23-H1 suppresses the expression of micro-RNA miR-660-5p [96]. Micro-RNA miR-660-5p has demonstrated ability to promote bone metastasis of lung cancer via regulation of the transcriptional regulator SMARCA5 [96]. Elucidation of the function of miR-660-5p reveals a network of transcriptional regulation via micro-RNAs and transcription factors operating within the bone metastasis of lung cancer.

#### 4.1.4. Renal Cell Carcinoma

Micro-RNAs play a key role in the development of RCC [97,98,99,100,101], however, despite this evidence, there have been few studies to date on micro-RNA expression specifically within bone metastasis of RCC. Micro-array-based quantification of micro-RNAs within matched primary RCC and bone metastatic samples from 57 patients identified a panel of 21 micro-RNAs, which demonstrated consistent down-regulation upon spread to bone [102]. The mechanism responsible for this micro-RNA down-regulation upon spread to bone appeared to be primarily epigenetic as treatment with trichostatin-A (a histone deacetylase—HDAC inhibitor) as well as 5-azacytosine (to reverse CpG methylation) was able to reverse these alterations in micro-RNA expression [102]. Predicted targets of this 21 micro-RNA panel were further validated and clustered within the “renal cell carcinoma” KEGG (Kyoto Encyclopaedia of Genes and Genomes) pathway [103].

### 4.2. Protein-Based Predictive Markers of Bone Metastasis

#### 4.2.1. Breast Cancer

Several studies point to the fact that the molecular composition of breast cancer cells at the protein level influences their risk of spreading to bone. In a study of molecular subtypes within breast cancer, immunohistochemical (IHC) staining for Ki67 was combined with profiling for human epidermal growth factor 2 (HER2) and oestrogen receptor (ER) demonstrating a higher rate of bone metastasis (87.8%) within ER-positive HER2 negative tumours with a Ki67 score > 13% compared to other subtypes [104].

Proteomic techniques have recently been applied to the identification of protein signatures predictive of bone-metastasis risk within breast cancer. Repeated intra-cardiac injection of fluorescently labelled triple-negative breast cancer cells (MDA-MB-231) with subsequent isolation of breast cancer cells from bone was used to generate a bone-homing cell-line (BM1) which homes to bone upon tail vein injection [105]. Proteomic comparison of the bone homing cell line to the parental MDA-MB-231 cells has identified several proteins with potential to be markers of bone metastasis risk including macrophage actin-capping protein-1 (CAPG) and PDZ domain-containing protein-1 (GIPC1) [21] as well as dedicator of cytokinesis-4 (DOCK4) [20,22] as upregulated specifically within bone-homing breast cancer cells. Validation of these markers by IHC within primary tumour cores from the large randomised phase-III AZURE trial demonstrated that high-level expression of both CAPG and GIPC1 correlated with the development of bone metastasis (*p* < 0.001) as well as with the efficiency of zoledronic acid in the prevention of skeletal metastases (*p* < 0.008) [21]. In a parallel analysis of DOCK4 levels within the AZURE trial, patients’ high DOCK4 expression correlated with the risk of bone metastasis (HR 2.13, 95%CI 1.06–4.30, *p* = 0.034) [22]. These proteins have considerable potential to not only predict the risk of breast cancer spread to bone but also influence patient treatment decisions. The mechanistic role of these proteins within bone metastasis of breast cancer has recently been elucidated with the discovery that CAPG acts as an epigenetic regulator for the expression of a key pro-metastatic regulator [106].

Targeted approaches have also been applied to look at proteins selected for a potential role within the spread to bone. Based upon a review of previous data a multi-protein panel was measured within bone homing breast cancer cells (including osteopontin, bone sialoprotein, CXCR4 and cadherin-11). Such studies demonstrated that the level of the chemokine C-C ligand 2 (CCL2) was inversely correlated with the risk of developing metastatic spread to bone.

Several studies have implicated proteins secreted from breast cancer cells that alter the activity of Wnt-family signalling within metastasis to bone. The Wnt inhibitor sclerostin (SOST) is secreted by breast cancer cells and acts to inhibit the differentiation of osteoblasts thus promoting osteolysis [107]. Additional Wnt family regulators are also highly expressed within bone metastasis including the Wnt-antagonist Dickopf-1 (DKK1), which has increased expression in breast cancer metastasis and promotes apoptosis of osteoblasts [108,109]. DKK1 expression is inhibited by nitrogen-containing bisphosphonates albeit at supra-physiological concentrations of the drug. DKK1 is upregulated within bone metastases of breast cancer as well as within serum from patients with bone metastasis [110].

Studies of the cell biology of breast cancer metastasis have identified key regulators of bone metastasis including nuclear p21-activated kinase-4 (nPAK4) a repressor of ERα-transactivation [111]. nPAK4 inhibits the metastasis repressor LIFR and thus facilitates breast cancer metastasis (including to bone). nPAK4 translocates to the nucleus of breast cancer cells together with ERα to achieve its effects. In addition to cellular levels of nPAK4, breast cancer cells which express high levels of RANK have a poorer disease-free survival within univariate analysis (*p* = 0.04) and multivariate analysis (*p* = 0.02) [112]. In the same study, RANKL expression was associated with an improved bone-metastasis DFS in multivariate analysis (*p* = 0.03) [112].

Additional circulating effectors of breast cancer spread to bone have been discovered including prolactin (PRL). High-level expression of PRL receptor on breast cancer cells is associated with shorter times to relapse (including relapse to bone) and this effect has been demonstrated to occur via the increased differentiation of osteoclasts and resultant increased bone lysis with the release of growth factors [113]. The circulating markers influencing bone metastasis are summarised in Table 2.

#### 4.2.2. Prostate Cancer

The high incidence of bone metastasis within advanced prostate cancer patients (occurring within 70–80% of patients with detectable metastases) has led to studies aiming to discover biomarkers predictive of bone spread. A systematic review of patient data (8644 patients) led to the proposal that a serum prostate-specific antigen (PSA) ≥ 20 ng/mL, a Gleason score ≥ 8 and the presence of locally advanced disease at the time of diagnosis was predictive of spread to bone [114]. The choice of these parameters is, however, the subject of debate and other published studies have contradicted these findings [115].

At the level of potential tissue-based markers of prostate cancer metastasis to bone, recent studies have compared the global proteome of bone metastatic prostate tumours post-surgical resection with primary prostate cancer tissue. These studies have identified molecular heterogeneity within prostate cancer bone metastases, with two predominant subgroups, one subtype expressing higher levels of targets of the androgen receptor (AR) as well as Golgi-resident and mitochondrial proteins, the other sub-group expressing high levels of proteins involved in DNA-repair and cell proliferation [116]. Expression of both of these molecular subtypes correlated with disease progression suggesting that this molecular heterogeneity should be considered when developing tissue-based biomarker panels predictive of bone metastasis risk [116].

Several studies have aimed to discover circulating biomarkers predictive for risk of bone metastasis in prostate cancer. Circulating tumour cells (CTCs) have become a key focus here with many studies seeking to address this correlation [117,118,119,120,121]. This has led to a definition of ≥5 CTC/7.5 mL as a cut-off above which men have an unfavourable prognosis. In patients, receiving abiraterone or chemotherapy a post-treatment decline in CTC count of ≥ 30% has been observed to correlate with better overall survival [122]. Molecular subtyping of prostate cancer CTCs has yielded information relevant to patient prognoses, including the observation that presence of epidermal growth factor receptor (EGFR) expressing CTCs in castration-resistant prostate cancer patients treated with docetaxel correlates with shorter overall survival (5.5 vs. 20.0 months for patients with no EGFR+ CTCs, *p* < 0.001) [123]. It has been observed that CTC counts are higher within patients with bone and visceral metastases (median 26, range 0–207), compared to patients with soft tissue lesions (median 0, range 0–28). Despite this, there is no correlation between CTC counts and parameters such as bone scan index (*p* = 0.81) and bone lesion count (*p* = 0.54) [124].

Secreted and circulating proteins predictive of prostate cancer bone metastasis have also been identified. The Wnt-inhibitor sclerostin is expressed at elevated levels within metastatic prostate cancer and appears to play a role in the promotion of osteolysis [125]. A significant positive relationship between sclerostin levels and bone turnover markers in metastatic PCa has been detected [126].

#### 4.2.3. Lung Cancer

The discovery of tissue-based biomarkers predictive of lung cancer metastasis to bone has involved both proteomic and immunohistochemical studies.

Immunohistochemical analysis of primary non-small cell lung cancer (NSCLC) samples, comparing tumours with associated bone metastases, to tumours with visceral metastases and non-metastatic tumours for expression of bone sialoprotein (BSP) demonstrated a significant correlation between BSP expression and the development of bone metastases (*p* < 0.001) as well as worse disease outcomes (*p* < 0.02) [127]. BSP was a component of a subsequent immunohistochemical study within stage III NSCLC samples utilising a four protein panel (BMP-4, CXCR4, osteopontin and BSP) demonstrating a predictive specificity of 66.7% and a sensitivity of 85.7% for the development of bone metastasis [127].

Proteomic analysis of primary lung adenocarcinoma (*n* = 9 patients) and bone metastases (*n* = 28 patients) identified a series of proteins differentially expressed upon the development of spread to bone [128]. Increased expression of enolase 1 (ENO1) (HR = 1.67, logrank *p* = 1.9 × 10^−5^), NME/NM23 nucleoside diphosphate kinase 2 (NME1-NME2) (HR = 2.65, logrank *p* = 3.9 × 10^−15^) and ribosomal protein lateral stalk subunit P2 (RPLP2) (HR = 1.77, logrank *p* = 2.9 × 10^−6^) were observed, and all were significantly associated with poor patient survival (*p* < 0.05) [128]. IHC staining of patient tissue sections demonstrated that high ENO1 staining (odds ratio, O.R. = 7.5, *p* = 0.034) and low-level staining of calcyphosine-1 CAPS1 (O.R. = 0.01, *p* < 0.0001) were associated with development of bone metastasis [128].

The discovery of circulating biomarkers of lung cancer bone metastasis has involved both proteomic approaches and the specifically targeted measurement of cytokine levels using immunological methods. In a retrospective study of circulating levels of PTHrP within 1149 hypercalcaemic lung cancer patients, levels of circulating PTHrP > 150 pmol/L were found to be associated with decreased median survival (1.4 months vs. 5.4 months) and bone metastasis incidence (12.5% vs. 71.4%) [129]. Wnt family regulators are also involved in the regulation of lung cancer spread to bone. Sclerostin-domain containing protein-1 (SOSTDC1) is downregulated within bone metastatic NSCLC, and it has been demonstrated that SOSTDC1 acts to inhibit cell-proliferation, migration and invasion of NSCLC cells [130]. Serum levels of the Wnt-inhibitor DKK1 have also been identified as elevated within patients with bone metastasis from NSCLC [131].

#### 4.2.4. Renal Cancer

There have been several studies aiming to identify the proteins driving the metastasis of RCC cells to bone. In one study, a bone-homing sub-clone of a clear cell RCC cell-line was generated by in vivo selection within a murine model [132,133]. Proteomic comparison of the osteotropic cell lines with the parental ccRCC cells identified 23 proteins which correlated with RCC metastasis and eight of these proteins were observed to be upregulated within bone metastasis tissue samples from RCC patients compared to primary RCC tumours [133]. These eight proteins were all involved in oxidative phosphorylation and mitochondrial function. Stress-induced phosphoprotein-1 (STIP1) was further characterised and found to exert two effects: (a) STIP1 promoted the proliferation and migration/invasion abilities of RCC tumour cells and (b) secreted STIP1 promoted the differentiation and activation of osteoclasts and the production of Cathepsin-K—a key enzyme involved in the degradation of the bone matrix [134].

Immunohistochemistry studies have demonstrated the key role of proteins such as the hepatocyte growth factor (HGF) receptor c-MET in the development of RCC bone metastases [135]. C-MET signalling has been implicated in the generation and maintenance of stem cells within bone metastases from RCC and use of a c-Met inhibitor was demonstrated to reduce the development of bone metastases within murine models and decrease the circulating serum level of the osteotropic factors IL-11 and CCL20. Inhibition of c-Met was accompanied by decreased osteoclast activity and an increase in osteoblast activity [135]. Circulating serum levels of CCL20 correlate with the development of bone metastases within RCC patients compared to metastasis to other sites [135].

### 4.3. Additional Sources of Circulating Markers

#### 4.3.1. Exosomes

Exosomes are small lipid vesicles 30–100 nm in diameter, which are generated within the endosomal compartment of cells and released into the extracellular space and circulation. Increasing evidence points to the fact that tumour-released exosomes can re-program cells within the pre-metastatic niche and thus be mechanistic agents of metastasis organotropism [136]. The organotropic properties of exosomes are determined by the proteins they express, including specific integrins [137]. Exosomes contain both proteins and micro-RNAs and as such, they can be key sources of both of these types of circulating biomarkers. A complete description of how exosomes act within bone metastatic cancers is beyond the scope of this review but it has been extensively described elsewhere [138,139].

#### 4.3.2. Circulating Tumour DNA (ctDNA)

Cancer cells are known to lyse and release DNA fragments into the circulation—termed circulating tumour DNA (ctDNA). Patients with metastatic tumours have higher levels of ctDNA than patients with localised tumours [140]. ctDNA has a wide variety of applications, and as an jkexample has been analysed within metastatic prostate cancer patients to reveal the development of key cancer driver mutations in a step towards personalised medicine [141]. ctDNA offers the possibility of a high sensitivity, PCR-based biomarker assay within bone metastasis (for a review see [142]).

#### 4.3.3. Circulating Tumour Cells (CTCs)

In addition to ctDNA, tumours also release tumour cells (termed circulating tumour cells—CTCs) into the bloodstream. CTCs are detected and measured using methods including the FDA-approved CellSearch system [143]. CTC counting has widespread utility. In a study of 276 patients with mCRPC, before starting docetaxel chemotherapy patients were stratified into “favourable” and “unfavourable” categories on the basis of CTC counts (<5 or ≥5 CTCs per 7.5 mL blood, respectively). Patients with “unfavourable” CTC counts at baseline had a worse median overall survival (11.5 months) compared to 21.7 months in patients with “favourable” counts (*p* < 0.0001) [117]. In addition to CTC counts, the molecular composition of CTCs, i.e., gene expression patterns, may also be informative in monitoring cancer spread. As an example, studies have looked at the relationship between ER/PR and HER2 status of CTCs in the prediction of breast cancer outcomes [144,145]. For a more comprehensive review of the utility of CTCs see [142].

As well as CTCs, bone metastatic cancers are also characterised by the presence of disseminated tumour cells (DTCs) which are released cancer cells present within the bone marrow that have entered a state of dormancy [146]. DTCs can be counted and isolated and recent studies have demonstrated that they can be subjected to techniques such as RNA-seq as well as genomic sequencing to detect the presence of key genetic alterations with a proven role in cancer cell spread to bone [147,148,149].

## 5. Towards Clinical Utility of Bone Metastasis Markers—Biomarker Panels and Spatial Information

As previously outlined, there have been numerous studies discovering potential biomarkers with the power to inform patient treatment decisions. Many of these studies have involved pre-clinical model systems (animal models and cell-lines) with some studies incorporating validation within patient-derived samples. A biomarker-based test for use within the clinic must deal with tumour heterogeneity and the diverse molecular subtypes within any given cancer, and thus biomarker panels have greater potential utility than individual markers (for example see [150]).

The process of bone metastasis has a spatial dimension not tested within many biomarker studies. Experiments conducted within animal models have demonstrated that osteoclast and osteoblast proliferation and number depended strongly on whether the cells were in close proximity and direct contact with metastatic breast cancer cells [151]. In view of this research, it is becoming apparent that the clinical utility of biomarkers within bone metastasis may depend both upon their spatial location within the bone metastatic process and the timing of their alteration, with early changes having greater potential for informing more timely interventions. Considerable research interest currently focuses on the pre-metastatic niche, a spatially distinct region of bone (which overlaps with the haematopoietic stem cell niche) where the early events of bone homing occur [152].

**Figure 3:**
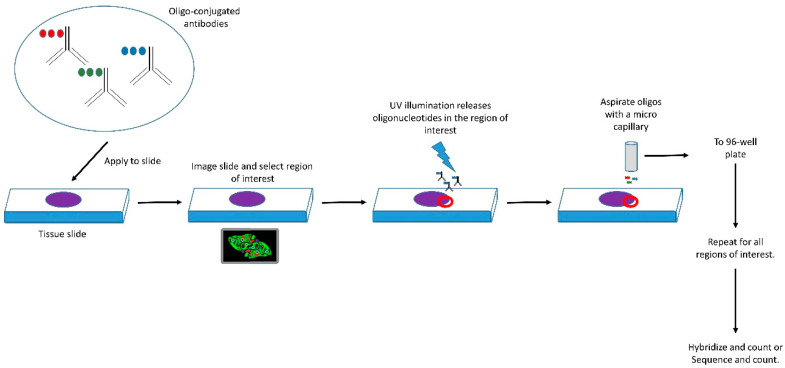
Digital spatial profiling (DSP) to reveal biomarker distribution within tissue sections.

Recent advances in biomarker imaging and quantification, include the development of techniques such as digital spatial profiling (DSP) [153]. DSP involves the use of oligonucleotide-barcoded antibodies or complementary-RNAs, which are coupled together via a UV-cleavable linker. Following application of the barcoded reagents to samples (which can include FFPE-tissue sections), UV laser illumination allows release and quantification of the oligonucleotides within spatially defined regions of the sample (typically 50–100 µm in diameter) and the quantification of binding using techniques such as an nCounter machine—see Figure 3. DSP technology enables the quantification of up to 96 individual micro-RNAs or proteins in biological samples with single-cell resolution [153]. Data from DSP can be normalised via the incorporation of a positive control reagent within the analysis, a detectable molecule which is known to have a uniform and constitutive expression (pan nuclear antigens are an example of the latter). The results of DSP analysis of protein targets are antibody-dependent and to aid morphological analysis of tissue sections a panel of standard intracellular organelle marker antibodies has been developed. Data analysis by DSP is an evolving and recent application and still progressing.

Digital spatial profiling uses hybridisation reagents with individually bar-coded detection reagents to measure the levels of panels of potential markers (either proteins or micro-RNAs) within spatially defined regions of tissue sections. The detection reagents are then applied to the tissue sections and then following tissue imaging, defined regions of interest can be selected. UV laser illumination within these regions of interest releases the bound oligonucleotide bar-code reagents, which are removed using a micro-capillary. These barcode oligonucleotide reagents are then quantified using methods such as an nCounter machine. The repeating of this process across different regions of interest within the tissue section defines the spatial distribution of the panel of markers. Normalisation of data can be achieved by incorporation of positive control detection reagents within the procedure.

Digital spatial profiling is a recently developed technology, which is beginning to shed light on the cellular mechanisms of bone metastasis. In a recent study of prostate cancer bone metastasis, DSP analysis identified unique immune cell populations and signalling within lytic and blastic types of prostate cancer. Blastic lesions were populated with immune cells enriched for pSTAT3 as well as components of the JAK-STAT pathway. In contrast, within lytic-type lesions, immune cells were enriched for pAKT activity as well as components of the PI3K-AKT pathway [154]. Immune checkpoint proteins including PD-L1, B7-H4, OX40L and IDO-1 were identified within blastic prostate cancer. It is clear from these pioneering studies that biopsy samples could guide the selection of patients into appropriate therapeutic interventions based on protein levels and micro-RNA expression of desired targets [154]. Just as molecular pathology has complemented the diagnosis, treatment, and management of primary tumours it is thus possible that it could also be successfully extended to patients with metastatic lesions.

The discovery and application of biomarker panels within bone metastasis of cancers is thus at an exciting juncture at which insights from cellular and molecular biology, and the ability to measure multiple biomarkers in a spatially distinct manner, can provide complementary information. Techniques like digital spatial profiling have the ability to quantify distinct functionally defined subsets of molecules and thus identify information of relevance towards biomarker discovery. In addition, future extension of this work will enable the identification of potential therapeutic targets within these biomarkers.

## 6. Summary

Bone metastasis of breast, prostate, lung and kidney cancer is a very significant complication within these disease types contributing towards increased patient mortality and reduced patient quality of life. As treatments for these cancer types improve, and patient survival times increase, bone metastasis from these tumours is destined to become an ever-increasing area requiring effective prognosis, detection and treatment. Advances in molecular profiling techniques have identified numerous biomarkers, encompassing genetic mutations, regulatory micro-RNAs and proteins that can inform patient treatment potentially informing personalised medicine initiatives. The prediction of those patients at greatest risk of developing bone metastasis will enable more prompt and timely employment of bone-directed therapeutic agents. In the example of breast cancer, treatment of bone metastasis involves the use of bisphosphonate drugs that can cause side effects such as osteonecrosis of the jaw (ONJ). In view of these side effects, biomarkers of bone metastasis risk could enable targeting of bisphosphonates to those patients at greatest risk of developing skeletal complications.

Metastatic spread to bone is a spatially defined process involving the priming of a pre-metastatic niche and the migration of incoming cancer cells towards a growth-supporting bone micro-environment. Recent advances in biomarker quantification and spatial imaging open up the possibility to probe the molecular alterations occurring at the active interface between metastatic cancer cells and the reactive stroma of the bone microenvironment. Multiplex biomarker quantification at the site of active metastasis initiation within bone will enable identification of the pivotal molecules differentially expressed at the tumour–stromal interface informing personalised medicine. In addition, there is considerable potential that molecules involved at the tumour–stromal interface will have utility within drug development initiatives within bone metastasis.

## 7. Conclusions

Metastatic spread to bone is one of the most severe complications arising from cancers of the breast, prostate, lung and kidney. Bone metastasis greatly reduces patient quality of life and decreases patient life expectancy. The combination of well-defined cell-line and animal based models, with recent developments in the ability to identify and quantify molecules within biological samples in a robust and medium-to-high throughput manner, is beginning to shed light on the essential processes driving cancer spread to bone. The key regulators responsible for the steps of cancer cell spread to bone and bone colonization have been identified leading to the discovery of novel drug targets and potential biomarkers of cancer spread. The recent ability to measure large panels of molecules within spatially well-defined regions of tumors with single-cell resolution will enable a study of the molecular alterations occurring at the invasive front of tumor cell metastasis.

Translation of these findings into improved patient treatments will require clinical validation of potential biomarkers within well-defined patient cohorts and subsequent biomarker measurement within large patient groups to insure that developed biomarker tests are robust. High throughput methods of biomarker quantification and validation, including targeted mass spectrometry, are beginning to bridge this gap. There is thus considerable potential for molecular analysis methods to improve patient treatment outcomes in the future.

## Figures and Tables

**Table 1:** Incidence of skeletal metastases within bone-metastatic cancers.

Incidence of Skeletal Metastases from Autopsy Studies			
		Incidence of Bone Metastases (%)	
Primary Tumour	Studies	Median	Range
Breast	5	73	47–85
Prostate	6	68	33–85
Thyroid	4	42	28–60
Kidney	3	35	33–40
Bronchus	4	36	30–55
Oesophagus	3	6	5–7
Gastrointestinal Tract	4	5	3–11
Rectum	3	11	8–13

Modified from Coleman-RE [1].

**Table 2:** Utility of bone turnover markers (BTMs) within bone metastasis.

Bone Turnover Marker	Application	Reference
**Bone Resorption Markers**		
N-Telopeptide of Type-I Collagen (NTX)	Diagnosis of bone metastasis—solid tumours	[57,58]
	Prognostic role for SRE development within bone metastases from solid tumours	[11]
	Prediction of treatment response in prostate cancer and breast cancer	[59,60]
	Prognostic role within bone targeting agents’ treatment	[59,61,62,63]
C-Telopeptide of Type-I Collagen (CTX)	Bone metastasis diagnosis within lung and prostate cancer	[57,58]
Tartrate Resistant Acid Phosphatase (TRACP)	Breast cancer bone metastasis—diagnosis of bone metastasis	[64,65]
Receptor Activator of Nuclear Factor ĸB-Ligand/Osteoprotegerin (RANK-L/OPG)	Breast cancer bone metastasis—diagnosis	[66]
Cross-Linked Carboxy-Terminal Telopeptide of Type-I Collagen (ICTP)	Lung cancer bone metastasis—diagnosis	[58]
Pyridinoline (PYD)	Breast cancer bone metastasis—diagnosis	[60]
**Bone Formation Markers**		
Pro-Collagen-Type I N-Terminal Pro-Peptide (P1NP)	Prostate/breast cancer bone metastasis—diagnosis	[57,64,65,66,67,68]
Pro-Collagen-Type I C-Terminal Pro-Peptide (P1CP)	Breast cancer bone metastasis—diagnosis	[57]
	Prostate cancer—prediction of treatment response	[60]
Bone Alkaline Phosphatase (BALP)	Diagnosis of bone metastasis—solid tumours	[65,69]
	Prognostic role for developing SRES within patients receiving bone targeting agents	[61,63,70]
	Prediction of treatment response in prostate cancer	[60]
	Prognostic role within bone metastases from solid tumours	[11]

## Supplementary Materials

The following are available online at https://www.mdpi.com/2072-6694/12/8/2109/s1, Table S1: Genomic and protein markers predictive of bone metastatic outcomes within bone-metastatic cancers.
