# Supplementary material for: Personal Medicine and Bone Metastases: Biomarkers, Micro-RNAs and Bone Metastases
Source: Cancers (Basel). 2020 Jul 29;12(8):2109. doi: 10.3390/cancers12082109 (PMC7465268; doi:10.3390/cancers12082109)
Supplement: Supplementary file 1 [file cancers-12-02109-s001.pdf]

Supplementary Materials: Personal Medicine and Bone Metastases: Biomarkers, Micro-RNAs and Bone Metastases

Steven L. Wood and Janet E. Brown

Table 1. Genomic and protein markers predictive of bone metastatic outcomes within bone-metastatic cancers.

Prostate Cancer			
**Genomic/functional genomic studies**			
**Study samples**	**Method used**	**Key findings**	**Reference**
Germline DNA analysed from patients with bone metastatic PCa.	Analysis using cancer SNP panel containing 1421 cancer-related SNPs.	14 candidate SNPs within 6 cancer-related genes (XRCC4, GATA3, PMS1, IL13, CASP8 and IGF1) correlates with disease survival.	[1]
PCa cell-lines DU145, LNCaP, PC-3 and PC-3M.	Methylation analysis of the promoter for BMP6.	Bone metastatic clones have promoter demethylation resulting in elevated BMP6 expression and bone homing.	[2]
Laser-capture and microdissected samples from PCa tumours, as well as analysis of the PCa cell-lines LNCaP and DU145.	Bisulphite sequencing and treatment of cells with demethylating agents.	ER-beta expression is high in normal prostate epithelium and bone metastases but low in grade 4/5 tumours, a phenomenon inversely correlated with ER-beta promoter methylation.	[3]
Prostate tissues from WT mice and mice with homozygour deletion of the HOXC6-gene.	Genome-wide localization analysis for HOXC6.	HOXC6 regulates both tumour promoters and suppressors related to bone metastasis including BMP7, FGFR2, IGFBP3 and PDGFRA.	[4]
DTCs from patients with organ confined diseasae and patients with advanced disease.	Array-comparative Genomic Hybridization.	Copy number changes (8p-loss, 8q-gain and androgen-receptor gene gain in metastatic patients.	[5]
PC3-prostate cancer cells isolated from bone.	Selective knockdown of CD44 and $\alpha v\beta 3$-integrin with expression analysis for RANKl.	Signalling via $\alpha v\beta 3$-integrin to SMAd5 and CD44 to RUNX2 both required for RANKL expression and osteoclastogenesis.	[6]
Bone metastatic samples PCa patients.	SNP-analysis focussing on genomic IGF-1 regulatory regions.	Three SNPS identified for IGF1 with significantly shorter overall survival time for patients with all three SNPs.	[7]
**Proteomic studies**			
**Study samples**	**Method used**	**Key findings**	**Reference**
Exosomes isolated from PCA patients with vertebral metastases.	2D-PAGE and MALDI-TOF-based protein identification.	Five proteins identified as being released within prostate derived exosomes including annexins-A1, A3 and A5 and dimethylarginine dimethylaminohydrolase.	[8]
Bone marrow aspirates from PCa patients.	Lectin enrichment, 2D-gel electrophoresis and mass-spectrometry.	Katanin-p60 is expressed in bone-metastatic PCa cells, along with basal-cell type markers p63 and	[9]

		high molecular weight cytokeratins.	
Pooled serum samples from BPH patients, patients with non-progressing PCa, patients with evidence of biochemical progression and patients with bone metastatic PCa.	iTRAQ analysis following serum-igh abundance depletion.	25 proteins identified which could distinguish progressing from non-progressing PCa. One protein eukaryotic transalation elongation factor 1 alpha (eEF1A1) validated by IHC as being elevated within osteoblasts adjacent to PCa tumour cells.	[10]
Conditioned media profiled from three PCa cell-lines - PC3 (bone metastasis), LNCaP (lymph-node metastasis) and 22Rv1 (prostate localized). Validation of markers performed using serum.	2D-LC and MSMS bottom-up analysis of conditioned media and serum samples.	Four proteins identified as potential PCa markers including; follistatin, pentraxin-3, spondin-2 and chemokine (C-X-C motif) ligand 16.	[11]
Urine samples from PCa patients and BPH-donors.	2D-PAGE based study.	Calgranulin-B/MRP-14 elevated within PCa urine compared to BPH urine.	[12]
Post-DRE urine samples from PCa and BPH donors.	MudPIT-shotgun proteomic analysis.	7 proteins identified which distinguish PCa from BPH.	[13]
Urine samples from PCa donors and healthy controls.	CE-MS.	Peptide panel which distinguishes PCa from healthy patients.	[14]
Urine samples from patients with benign lesions and PCa.	SELDI-TOF-MS.	72-peak peptide panel which distinguishes PCa from benign conditions.	**[15]**
Urine samples from PCA patients	ICAT-labelling and MSMA analysis.	Presence of CD90 within PCa urine with elevated CD90/Thy-1 in PCa urine. CD90 detected following collagenase digestion of PCA tissues.	**[16]**
		**Breast Cancer**	
**Genomic/functional genomic studies**			
**Study samples**	**Method used**	**Key findings**	**Reference**
Epithelial cells from primary breast tumours, as well as metastases to bone, brain and lung.	Methylation-specifc PCCR with validation by IHC.	Hypermethylation silences HIN-1 and RARβ within BCa bone metastases.	[17]
FFPE tumour sections from BCa patients treated with trastazumab	FISH and SNP genotyping.	Mutations within PIK3Ca and loss of PTEN correlate with shorter survival times and reduced efficiacy of treatment.	[18]
Breast tumour-cells lines cultured +/- DNA-demthylating agents.	RT-PCR and IHC validation.	Differentiation-related-gene (Drg-1) is silenced by promoter methylation within highly metastatic BCa cell-lines.	[19]
Bone marrow micro-metastatic cells from breast cancer patients.	Array-CGH.	DNA amplifications as small as 4.4-5 MBytes within bone marrow micrometastases.	[20]
Bone-homing breast cancer cell-lines.	Electrophoretic Mobility Shift Assays (EMSA).	Promoter methylation for E-cadherin and WWdomain-containing oxidoreductase (Wwox) within bone metastatic cell-lines.	[21]

mRNA-profiling during breast cancer metastasis.	Array-based profiling.	Down-regulation of miR-335 during bone metastasis. miR-335 targets SOX4 and tenascin. Correlation with risk of relapse.	[22]
Breast cancer metastatic cells.	cDNA-Microarray	Essential role for Src and AKT pathways within survival of BCa metastatic cells.	[23]
MDA-MB-231 cell-line and variants with increasing degrees of bone homing ability.	MSP and bisulphite sequencing and cDNA-microarray.	Bone homing ability correlates with differential expression of 128-protein panel including cystatin-E/M (CST6).	[24]
Primary breast tumours	cDNA-microarray.	70-gene signature identified which predicts poor-prognosis, hazard ration for presence of signature 5.1.	[25]
Breast cancer cell-line panel including MDA-MB-231.	qRT-PCR.	Upregulation of miR-224 with resulting down-regulation of RKIP expression and de-represpression of MMP1, CXCR4 and OPG.	[26]
Publicly available gene-expression datasets from breast cancer metastasis studies.	Computational analysis of gene expression datasets.	SMAD4 and HIF1 identified as regulators of bone metastasis, BACH1 identified as a master regulator of bone-homing.	[27]
Breast cancer metastases to bone, brain and lung.	cDNA-microarray analysis with network-based classification analysis.	A protein-protein network identified with metastasis to bone mainly involving immune-response proteins.	[28]
**Proteomic studies**			
**Study samples**	**Method used**	**Key findings**	**Reference**
MCF-7/*neo* and MCF-7/*Her2* over-expressing cell-lines,	Cytokine-Antibody Arrays.	GRO and IL-8 expression levsl alter in response to HER2 over-expression. Treatment with the TKI-gefitinib reverses these alterations.	[29]
Breast tumour derived cell-lines and tissue microarrays.	SILAC-MS followed by validation by IHC of TMAs.	Protein-expression signature predictive of breast cancer stage within ER-negative BCa. Signature validated by TMA studies.	[30]
MDA-MB-231 cell-line and bone homing variants.	LFQ-MS.	128-proteins identified as differentially expressed during development of bone-homing, including Cystatin-E/M (CST6).	[24]
Co-cultured MDA-MB-231 cells and osteoblast cell-line.	iTRAQ	Increased expression of MMP13 by osteoblasts in response to oncostatin-M and SAA3 secretion by breast cancer cells.	[31]
Invasive breast cancer specimens.	Tissue Arrays.	Elevated levels of MMP -2, -7 and -14 and TIMP-3 and low expression of MMP-9 are predictive of breast cancer metastasis.	[32]
Breast cancer cell line series from low-grade through to high-grade.	iTRAQ	Warburg-effect proteins elevated as well as altered expression of adenylate-kinase-1, copper-transport-protein ATOX1 and histone H2B type 1M.	[33]

Breast cancer section TMAs.	TMA-analysis	Identification of MMP1 and ADAMTS1 as active enzymes within the release of soluble EGF-like ligands promoting breast cancer metastasis.	[34]
Primary breast cancer specimens.	IHC within TMAs.	Altered expression of serpins, laminin chains and MMPs within subclasses of tumours correlating with altered invasiveness. elevated levels of integrins and metalloproteases as well as reduced expression of laminins correlates with poor prognosis.	[35]
	**Lung Cancer**		
**Genomic/functional genomic studies**			
**Study samples**	**Method used**	**Key findings**	**Reference**
Analysis of lung cancer cell-lines.	qRT-PCR analysis.	The Nm23-H1 gen suppresses expression of micro-RNA. miR-660-5p regulates metastasis via regulation of SMARCA5.	[36]
Lung cancer samples from 8 NSCLC patients with linked data relating to bone metastasis occurrence.	Next Generation Sequencing (NGS)	Bone metastasis associated with fibroblast growth factor receptor – (FGFR), ataxia telangiectasia mutated (ATM), and cyclin dependent kinase-12 (CDK12) expression.	[37]
**Proteomic studies**			
**Study samples**	**Method used**	**Key findings**	**Reference**
NSCLC primary tumours and paired bone metastasis samples,	IHC analysis.	(BMP-4, CXCR4, osteopontin levels predict lung cancer metastasis to bone.	**[38]**
Patient-derived lung adenocarcinoma and bone metastasis sections	2D-gel electrophoresis and mass-spectrometry.	Increased expression of enolase 1 - ENO1, NME/NM23 nucleoside diphosphate kinase 2 (NME1-NME2) and ribosomal protein lateral stalk subunit P2 (RPLP2) in poor survival patients.	**[39]**
Lung adenocarcinoma patient serum.	ELISA assay.	PTHrP levels predict bone metastatic outcomes, hyper-calcaemia and survival.	**[40]**
NSCLC serum samples.	ELISA assay.	Sclerostin levels downregulated within bone metastasis.	**[41]**
NSCLC serum samples.	ELISA assay.	DKK1 levels elevated within serum of bone metastatic patients.	**[42]**
	**Renal Cell Carcinoma (RCC)**		
**Genomic/functional genomic studies**			
**Study samples**	**Method used**	**Key findings**	**Reference**
RCC primary tumours and patient samples from metastatic sites.	Next Generation Sequencing (NGS).	Mutations within the SETD2 (SET Domain Containing 2, Histone Lysine Methyltransferase in RCC bone metastasis.	[43]
**Proteomic Studies**			
Study samples	Method used	Key findings	Reference
RCC cell-lines and a bone homing variant cell line.	LC-MS analysis.	Eight proteins involved in mitochondrial metabolism altered in bone homing cells.	[44]
Patient derived samples (tissues and serum).	IHC (tissues) and ELISA (serum).	c-MET and CCL20 levels correlate with bone metastasis.	[45]

Reference

1. Tsuchiya, N.; Narita, S.; Inoue, T.; Saito, M.; Numakura, K.; Huang, M.; Hatakeyama, S.; Satoh, S.; Saito, S.; Ohyama, C.; et al. Insulin-like growth factor-1 genotypes and haplotypes influence the survival of prostate cancer patients with bone metastasis at initial diagnosis. *BMC Cancer* **2013**, *13*, 150, doi:10.1186/1471-2407-13-150.
2. Tamada, H.; Kitazawa, R.; Gohji, K.; Kitazawa, S. Epigenetic Regulation of Human Bone Morphogenetic Protein 6 Gene Expression in Prostate Cancer. *J. Bone Miner. Res.* **2001**, *16*, 487–496, doi:10.1359/jbmr.2001.16.3.487.
3. Zhu, X.; Leav, I.; Leung, Y.-K.; Wu, M.; Liu, Q.; Gao, Y.; McNeal, J.E.; Ho, S.-M. Dynamic Regulation of Estrogen Receptor-β Expression by DNA Methylation During Prostate Cancer Development and Metastasis. *Am. J. Pathol.* **2004**, *164*, 2003–2012, doi:10.1016/s0002-9440(10)63760-1.
4. McCabe, C.D.; Spyropoulos, D.D.; Martin, D.; Moreno, C.S. Genome-wide analysis of the homeobox C6 transcriptional network in prostate cancer. *Cancer Res.* **2008**, *68*, 1988–1996, doi:10.1158/0008-5472.CAN-07-5843.
5. Holcomb, I.N.; Grove, D.I.; Kinnunen, M.; Friedman, C.L.; Gallaher, I.S.; Morgan, T.M.; Sather, C.L.; Delrow, J.J.; Nelson, P.S.; Lange, P.H.; et al. Genomic alterations indicate tumor origin and varied metastatic potential of disseminated cells from prostate cancer patients. *Cancer Res.* **2008**, *68*, 5599–5608, doi:10.1158/0008-5472.CAN-08-0812.
6. Gupta, A., Cao, W. and Chellaiah, M.A. Integrin alphavbeta3 and CD44 pathways in metastatic prostate cancer cells support osteoclastogenesis via a Runx2/Smad 5/receptor activator of NF-kappaB ligand signaling axis. *Mol. Cancer*, **2012**, *11*, 66.
7. Tsuchiya, N.; Matsui, S.; Narita, S.; Kamba, T.; Mitsuzuka, K.; Hatakeyama, S.; Horikawa, Y.; Inoue, T.; Saito, S.; Ohyama, C.; et al. Distinct Cancer-Specific Survival in Metastatic Prostate Cancer Patients Classified by a Panel of Single Nucleotide Polymorphisms of Cancer-Associated Genes. *Genes Cancer* **2013**, *4*, 54–60, doi:10.1177/1947601913481354.
8. Ronquist, G.; Ronquist, G.; Larsson, A.; Carlsson, L. Proteomic analysis of prostate cancer metastasis-derived prostasomes. *Anticancer. Res.* 2010, *30*, 285–90.
9. Ye, X.; Lee, Y.-C.; Choueiri, M.; Chu, K.; Huang, C.-F.; Tsai, W.-W.; Kobayashi, R.; Logothetis, C.J.; Yu-Lee, L.-Y.; Lin, K.-M. Aberrant expression of katanin p60 in prostate cancer bone metastasis. *Prostate* **2011**, *72*, 291–300, doi:10.1002/pros.21431.
10. Rehman, I., Evans, C.A.; Glen, A.; Cross, S.S.; Eaton, C.L.; Down, J.; Pesce, G.; Phillips, J.T.; Yen, S.W.; Thalmann, G.N.; et al. iTRAQ identification of candidate serum biomarkers associated with metastatic progression of human prostate cancer. *PLoS One*, **2012**, *7*, e30885.
11. Sardana, G.; Jung, K.; Stephan, C.; Diamandis, E.P. Proteomic Analysis of Conditioned Media from the PC3, LNCaP, and 22Rv1 Prostate Cancer Cell Lines: Discovery and Validation of Candidate Prostate Cancer Biomarkers. *J. Proteome Res.* **2008**, *7*, 3329–3338, doi:10.1021/pr8003216.
12. Rehman, I.; Azzouzi, A.; Catto, J.W.; Allen, S.; Cross, S.; Feeley, K.; Meuth, M.; Hamdy, F. Proteomic analysis of voided urine after prostatic massage from patients with prostate cancer: A pilot study. *Urology* **2004**, *64*, 1238–1243, doi:10.1016/j.urology.2004.06.063.
13. Principe, S.; Kim, Y.; Fontana, S.; Ignatchenko, V.; Nyalwidhe, J.O.; Lance, R.S.; Troyer, D.A.; Alessandro, R.; Semmes, O.J.; Kislinger, T.; et al. Identification of Prostate-Enriched Proteins by In-depth Proteomic Analyses of Expressed Prostatic Secretions in Urine. *J. Proteome Res.* **2012**, *11*, 2386–2396, doi:10.1021/pr2011236.
14. Theodorescu, D.; Schiffer, E.; Bauer, H.W.; Douwes, F.; Eichhorn, F.; Polley, R.; Schmidt, T.; Schöfer, W.; Zürbig, P.; Good, D.M.; et al. Discovery and validation of urinary biomarkers for prostate cancer. *Proteom. Clin. Appl.* **2008**, *2*, 556–570, doi:10.1002/prca.200780082.
15. Okamoto, A.; Yamamoto, H.; Imai, A.; Hatakeyama, S.; Iwabuchi, I.; Yoneyama, T.; Hashimoto, Y.; Koie, T.; Kamimura, N.; Mori, K.; et al. Protein profiling of post-prostatic massage urine specimens by surface-enhanced laser desorption/ionization time-of-flight mass spectrometry to discriminate between prostate cancer and benign lesions. *Oncol. Rep.* 2009, *21*, 73–79.
16. True, L.D.; Zhang, H.; Ye, H.; Huang, C.-Y.; Nelson, P.S.; von Haller, P.D.; Tjoelker, L.W.; Kim, J.-S.; Qian, W.-J.; Smith, R.D.; et al., CD90/THY1 is overexpressed in prostate cancer-associated fibroblasts and could serve as a cancer biomarker. *Mod. Pathol.* **2010**, *23*, 1346–1356.

17. Mehrotra, J.; Vali, M.; McVeigh, M.; Kominsky, S.L.; Fackler, M.J.; Lahti-Domenici, J.; Polyak, K.; Sacchi, N.; Garrett-Mayer, E.; Argani, P.; et al. Very high frequency of hypermethylated genes in breast cancer metastasis to the bone, brain, and lung. *Clin. Cancer Res.* **2004**, *10*, 3104–3109, doi:10.1158/1078-0432.ccr-03-0118.
18. Razis, E.; Bobos, M.; Kotoula, V.; Eleftheraki, A.; Kalofonos, H.; Pavlakis, K.; Papakostas, P.; Aravantinos, G.; Rigakos, G.; Efstratiou, I.; et al. Evaluation of the association of PIK3CA mutations and PTEN loss with efficacy of trastuzumab therapy in metastatic breast cancer. *Breast Cancer Res. Treat.* **2011**, *128*, 447–456, doi:10.1007/s10549-011-1572-5.
19. Bandyopadhyay, S.; Pai, S.K.; Hirota, S.; Hosobe, S.; Takano, Y.; Saito, K.; Piquemal, D.; Commes, T.; Watabe, M.; Gross, S.C.; et al. Role of the putative tumor metastasis suppressor gene Drg-1 in breast cancer progression. *Oncogene* **2004**, *23*, 5675–5681, doi:10.1038/sj.onc.1207734.
20. Fuhrmann, C.; Schmidt-Kittler, O.; Stoecklein, N.H.; Petat-Dutter, K.; Vay, C.; Bockler, K.; Reinhardt, R.; Ragg, T.; Klein, C.A. High-resolution array comparative genomic hybridization of single micrometastatic tumor cells. *Nucleic Acids Res.* **2008**, *36*, e39, doi:10.1093/nar/gkn101.
21. Matteucci, E.; Maroni, P.; Luzzati, A.; Perrucchini, G.; Bendinelli, P.; Desiderio, M.A. Bone metastatic process of breast cancer involves methylation state affecting E-cadherin expression through TAZ and WWOX nuclear effectors. *Eur. J. Cancer* **2013**, *49*, 231–244, doi:10.1016/j.ejca.2012.05.006.
22. Tavazoie, S.F.; Alarcón, C.; Oskarsson, T.; Padua, D.; Wang, Q.; Bos, P.D.; Gerald, W.L.; Massagué, J. Endogenous human microRNAs that suppress breast cancer metastasis. *Nature* **2008**, *451*, 147–152, doi:10.1038/nature06487.
23. Zhang, X.H.-F.; Wang, Q.; Gerald, W.; Hudis, C.A.; Norton, L.; Smid, M.; Foekens, J.A.; Massagué, J. Latent Bone Metastasis in Breast Cancer Tied to Src-Dependent Survival Signals. *Cancer Cell* **2009**, *16*, 67–78, doi:10.1016/j.ccr.2009.05.017.
24. Jin, L.; Zhang, Y.; Li, H.; Yao, L.; Fu, D.; Yao, X.; Xu, L.X.; Hu, X.; Hu, G. Differential secretome analysis reveals CST6 as a suppressor of breast cancer bone metastasis. *Cell Res.* **2012**, *22*, 1356–1373, doi:10.1038/cr.2012.90.
25. Van De Vijver, M.J.; He, Y.D.; Veer, L.J.V. 'T; Dai, H.; Hart, A.A.; Voskuil, D.W.; Schreiber, G.J.; Peterse, J.L.; Roberts, C.; Marton, M.J.; et al. A Gene-Expression Signature as a Predictor of Survival in Breast Cancer. *New Engl. J. Med.* **2002**, *347*, 1999–2009, doi:10.1056/nejmoa021967.
26. Huang, L.; Dai, T.; Lin, X.; Zhao, X.; Chen, X.; Wang, C.; Li, X.; Shen, H.-Y.; Wang, X. MicroRNA-224 targets RKIP to control cell invasion and expression of metastasis genes in human breast cancer cells. *Biochem. Biophys. Res. Commun.* **2012**, *425*, 127–133, doi:10.1016/j.bbrc.2012.07.025.
27. Liang, Y.; Wu, H.; Lei, R.; Chong, R.A.; Wei, Y.; Lu, X.; Tagkopoulos, I.; Kung, S.-Y.; Yang, Q.; Hu, G.; et al. Transcriptional Network Analysis Identifies BACH1 as a Master Regulator of Breast Cancer Bone Metastasis. *J. Boil. Chem.* **2012**, *287*, 33533–33544, doi:10.1074/jbc.M112.392332.
28. Jahid, J.; Ruan, J. A Steiner tree-based method for biomarker discovery and classification in breast cancer metastasis. *BMC Genom.* **2012**, *13*, S8, doi:10.1186/1471-2164-13-S6-S8.
29. Vázquez-Martín, A.; Colomer, R.; Menendez, J.A. Protein array technology to detect HER2 (erbB-2)-induced 'cytokine signature' in breast cancer. *Eur. J. Cancer* **2007**, *43*, 1117–1124, doi:10.1016/j.ejca.2007.01.037.
30. Geiger, T.; Madden, S.F.; Gallagher, W.M.; Cox, J.; Mann, M. Proteomic Portrait of Human Breast Cancer Progression Identifies Novel Prognostic Markers. *Cancer Res.* **2012**, *72*, 2428–2439, doi:10.1158/0008-5472.can-11-3711.
31. Morrison, C.; Mancini, S.; Cipollone, J.; Kappelhoff, R.; Roskelley, C.; Overall, C.M. Microarray and Proteomic Analysis of Breast Cancer Cell and Osteoblast Co-cultures. *J. Biol. Chem.* **2011**, *286*, 34271–34285, doi:10.1074/jbc.m111.222513.
32. Del Casar, J.M.; Alvarez, E.; Junquera, S.; Marin, L.; Bongera, M.; Vazquez, J.; Vizoso, F.J.; González, L.O.; González, L. Comparative analysis and clinical value of the expression of metalloproteases and their inhibitors by intratumor stromal fibroblasts and those at the invasive front of breast carcinomas. *Breast Cancer Res. Treat.* **2009**, *116*, 39–52, doi:10.1007/s10549-009-0351-z.
33. Choong, L.Y.; Lim, S.; Chong, P.K.; Wong, C.Y.; Shah, N.; Lim, Y.P. Proteome-Wide Profiling of the MCF10AT Breast Cancer Progression Model. *PLOS ONE* **2010**, *5*, e11030, doi:10.1371/journal.pone.0011030.
34. Lü, X.; Wang, Q.; Hu, G.; Van Poznak, C.; Fleisher, M.; Reiss, M.; Massagué, J.; Kang, Y. ADAMTS1 and MMP1 proteolytically engage EGF-like ligands in an osteolytic signaling cascade for bone metastasis. *Genes Dev.* **2009**, *23*, 1882–1894, doi:10.1101/gad.1824809.

35. Bergamaschi, A.; Tagliabue, E.; Sørlie, T.; Naume, B.; Triulzi, T.; Orlandi, R.; Russnes, H.; Nesland, J.; Tammi, R.; Auvinen, P.; et al. Extracellular matrix signature identifies breast cancer subgroups with different clinical outcome. *J. Pathol.* **2007**, *214*, 357–367, doi:10.1002/path.2278.
36. Ai, C.; Ma, G.; Deng, Y.; Zheng, Q.; Gen, Y.; Li, W.; Li, Y.; Zu, L.; Zhou, Q. Nm23-H1 inhibits lung cancer bone-specific metastasis by upregulating miR-660-5p targeted SMARCA5. *Thorac. Cancer* **2020**, *11*, 640–650, doi:10.1111/1759-7714.13308.
37. Hong, W.; Zhang, K.; Zhang, M.; Zhu, J. Screening of gene mutations associated with bone metastasis in nonsmall cell lung cancer. *J. Cancer Res. Ther.* **2016**, *12*, 186, doi:10.4103/0973-1482.200597.
38. Papotti, M.; Kalebic, T.; Volante, M.; Chiusa, L.; Bacillo, E.; Cappia, S.; Lausi, P.O.; Novello, S.; Borasio, P.; Scagliotti, G.V. Bone Sialoprotein Is Predictive of Bone Metastases in Resectable Non–Small-Cell Lung Cancer: A Retrospective Case-Control Study. *J. Clin. Oncol.* **2006**, *24*, 4818–4824, doi:10.1200/jco.2006.06.1952.
39. Yang, M.; Sun, Y.; Sun, J.; Wang, Z.; Zhou, Y.; Yao, G.; Gu, Y.; Zhang, H.; Zhao, H. Differentially expressed and survival-related proteins of lung adenocarcinoma with bone metastasis. *Cancer Med.* **2018**, *7*, 1081–1092, doi:10.1002/cam4.1363.
40. Hiraki, A.; Ueoka, H.; Bessho, A.; Segawa, Y.; Takigawa, N.; Kiura, K.; Eguchi, K.; Yoneda, T.; Tanimoto, M.; Harada, M. Parathyroid hormone-related protein measured at the time of first visit is an indicator of bone metastases and survival in lung carcinoma patients with hypercalcemia. *Cancer* **2002**, *95*, 1706–1713, doi:10.1002/cncr.10828.
41. Chen, G.; Gong, H.; Wang, T.; Wang, J.; Han, Z.; Bai, G.; Han, S.; Yang, X.; Zhou, W.; Liu, T.; et al. SOSTDC1 inhibits bone metastasis in non-small cell lung cancer and may serve as a clinical therapeutic target. *Int. J. Mol. Med.* **2018**, *42*, 3424–3436, doi:10.3892/ijmm.2018.3926.
42. Qiao, R.; Zhong, R.; Chang, Q.; Teng, J.; Pei, J.; Han, B.; Chu, T. Serum dickkopf-1 as a clinical and prognostic factor in non-small cell lung cancer patients with bone metastases. *Oncotarget* **2017**, *8*, 79469–79479, doi:10.18632/oncotarget.18446.
43. Becerra, M.F.; Reznik, E.; Redzematovic, A.; Tennenbaum, D.; Kashan, M.; Ghanaat, M.; Casuscelli, J.; Manley, B.; Jonsson, P.; DiNatale, R.G.; et al. Comparative Genomic Profiling of Matched Primary and Metastatic Tumors in Renal Cell Carcinoma. *Eur. Urol. Focus* **2018**, *4*, 986–994, doi:10.1016/j.euf.2017.09.016.
44. Wang, J.; Zhao, X.; Qi, J.; Yang, C.; Cheng, H.; Ren, Y.; Huang, L. Eight proteins play critical roles in RCC with bone metastasis via mitochondrial dysfunction. *Clin. Exp. Metastasis* **2015**, *32*, 605–622, doi:10.1007/s10585-015-9731-4.
45. D'Amico, L.; Belisario, D.; Migliardi, G.; Grange, C.; Bussolati, B.; D'Amelio, P.; Perera, T.; Dalmasso, E.; Carbonare, L.D.; Godio, L.; et al. C-met inhibition blocks bone metastasis development induced by renal cancer stem cells. *Oncotarget* **2016**, *7*, 45525–45537, doi:10.18632/oncotarget.9997.
